# Author Correction: The Oligodendrocyte Transcription Factor 2 OLIG2 regulates transcriptional repression during myelinogenesis in rodents

**DOI:** 10.1038/s41467-022-30945-w

**Published:** 2022-06-01

**Authors:** Kunkun Zhang, Shaoxuan Chen, Qihua Yang, Shuanghui Guo, Qiang Chen, Zhixiong Liu, Li Li, Mengyun Jiang, Hongda Li, Jin Hu, Xu Pan, Wenbo Deng, Naian Xiao, Bo Wang, Zhan-xiang Wang, Liang Zhang, Wei Mo

**Affiliations:** 1 Department of Neurosurgery and Department of Neuroscience, the First Affiliated Hospital of Xiamen University, State Key Laboratory of Cellular Stress Biology, School of Medicine, Xiamen University, Fujian, China; 2 Xiamen Key Laboratory of Brain Center, the First Affiliated Hospital of Xiamen University, Fujian, China; 3 School of Life Sciences, Innovation Center for Cell Signaling Network, Xiamen University, Fujian, China; 4 School of Pharmaceutical Sciences, Xiamen University, Xiamen, 361102 Fujian, China

**Keywords:** Epigenetics in the nervous system, Myelin biology and repair, Cellular neuroscience

Correction to: *Nature Communications* 10.1038/s41467-022-29068-z, published online 17 March 2022.

The original version of this Article contained an error in the ‘Animals’ section of the Methods, which incorrectly read ‘*Cnp-Cre, Olig2*^*Flox/Fox*^ and *Sox11*^*Flox/Flox*^ mice were generously provided by Dr. Luis F. Parada (Department of Developmental Biology, University of Texas Southwestern Medical School, Dallas, TX, USA).’ The correct version adds ‘Cnp-Cre (generated by Dr. Klaus Nave), *Olig2*^*Flox/Fox*^ and *Sox11*^*Flox/Flox*^ mice were generously provided by Dr. Luis F. Parada (Department of Developmental Biology, University of Texas Southwestern Medical School, Dallas, TX, USA).’ This has been corrected in both the PDF and HTML versions of the Article.

