# Supplementary material for: The Oligodendrocyte Transcription Factor 2 OLIG2 regulates transcriptional repression during myelinogenesis in rodents
Source: Nat Commun. 2022 Mar 17;13:1423. doi: 10.1038/s41467-022-29068-z (PMC8931116; doi:10.1038/s41467-022-29068-z)
Supplement: Supplementary file 1 — Supplymentary Information [file 41467_2022_29068_MOESM1_ESM.pdf]

Supplementary Information

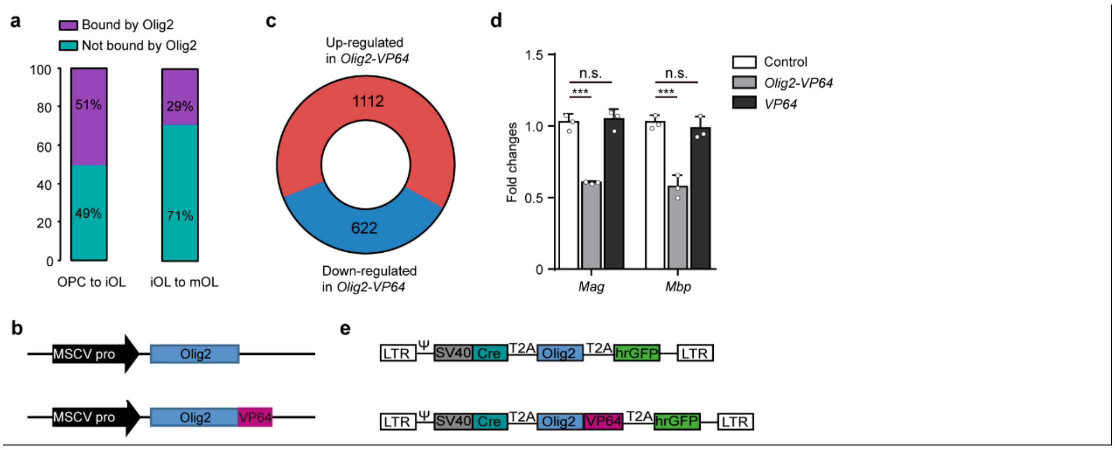

Supplementary Figure 1. The repressor role of OLIG2 is essential for oligodendrocyte differentiation

(**a**) Box plots showing the percentage of downregulated genes, bound by OLIG2 in rat iOLs, during OPC differentiation or oligodendrocyte maturation. (**b**) Schematic diagram showing the strategy that we transfected OLIG2 or OLIG2-VP64 in rat OPCs. (**c**) Pie chart showing number of genes that were significantly upregulated or downregulated in OLIG2-VP64 transfected rat iOLs. (**d**) Real-time PCR analysis of *Mag* and *Mbp* in rat OPCs under differentiation condition following transfection with vehicle, *Olig2-VP64* or *VP64*. Error bars indicate SEM (***$p<0.001$. one-way ANOVA). (**e**) Schematic diagram showing the retrovirus that we injected in mouse brain.

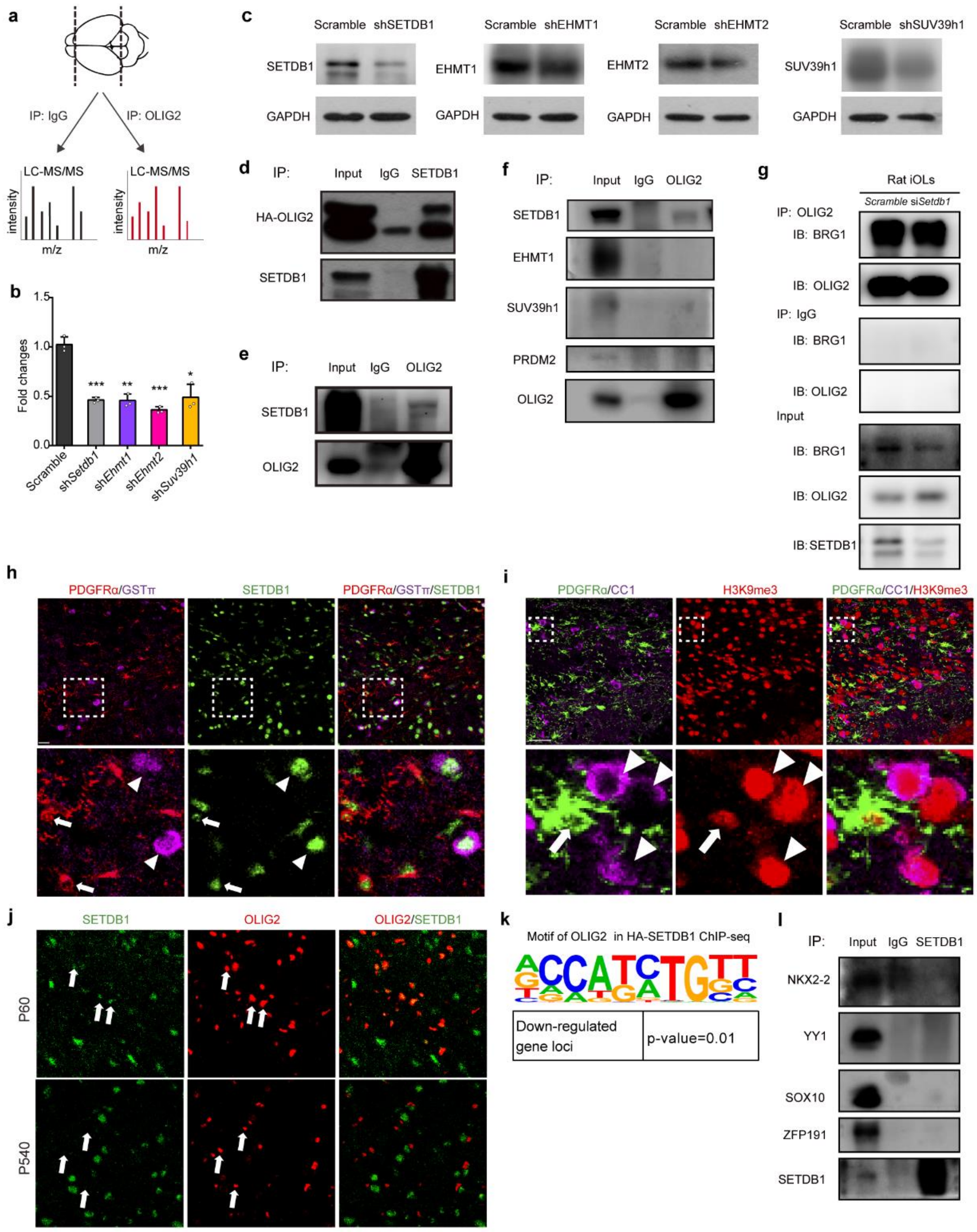

Supplementary Figure 2. OLIG2 assembles a repressor complex to dominant oligodendrocyte differentiation

(**a**) Schematic illustration of OLIG2 binding protein screening. (**b-c**) Efficiency of H3K9me transferases (Setdb1, Ehmt1, Ehmt2 and Suv39h1) knockdown in rat OPCs was examined by quantitative real-time PCR (b) Error bars indicate SEM (***p<0.001; sh*Ehmt1*, p=0.0062; sh*Suv39h1*, p=0.015 one-way ANOVA) and Western Blotting (c). n=3 independent experiments. (**d-e**) Co-immunoprecipitation (Co-IP) of HA-OLIG2 with endogenous SETDB1 from transiently transfected 293T cells (d) or brain tissue lysate of P5 pups (e). n=3 independent experiments. (**f**) Co-IP of OLIG2 with HMTs from rat iOLs. n=3 independent experiments. (**g**) Co-IP of OLIG2 with BRG1 from control or *Setdb1* knock-down rat iOLs. n=3 independent experiments. (**h**) Brain at P14 was immunostained with GSTπ, PDGFRα and SETDB1. Boxed areas in upper panels are shown at

a high magnification in corresponding lower panels. Arrow indicates the co-expression of SETDB1 with PDGFRα, and arrowhead indicates the co-expression of SETDB1 with GSTπ. n=3 different mice. (**i**) Brain at P14 was immunostained with PDGFRα, CC1 and H3K9me3. Boxed areas in upper panels are shown at a high magnification in lower panels. Arrow indicates the co-expression of H3K9me3 with PDGFRα, and arrowhead indicates the co-expression of H3K9me3 with CC1. n=3 different mice. (**j**) Brain at P60 or P540 was immunostained with SETDB1 and OLIG2. n=3 different mice. (**k**) OLIG2 binding motif was identified by HOMER analysis within HA-SETDB1 peaks among loci of genes downregulated upon rat OPC differentiation. Letter size indicates nucleotide frequency at each position of OLIG2 binding motif. (**l**) Co-IP of indicated transcriptional factors with SETDB1 from rat iOLs. n=3 independent experiments. Scale bars: 50μm in (**g**-**i**).

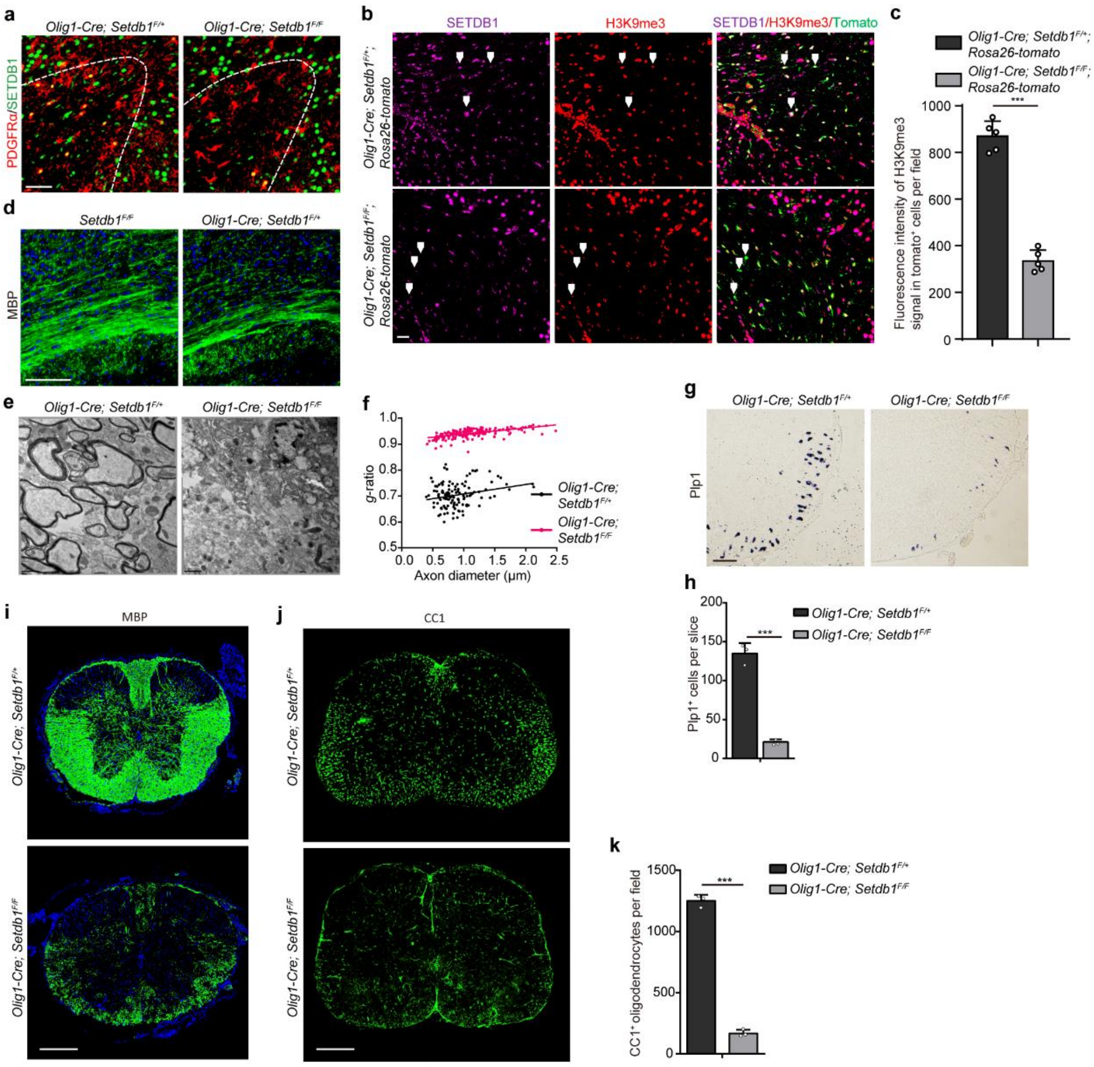

Supplementary Figure 3. SETDB1 is required for oligodendrocyte differentiation in spinal cord

(**a**) Brain sections from *Olig1-Cre*; *Setdb1*$^{F/+}$ and *Olig1-Cre*; *Setdb1*$^{F/F}$ mice at P14 were immunostained with PDGFRα and SETDB1. n=3 control and mutant mice. (**b**) Immunostaining of SETDB1, H3K9me3 and Rosa26-tomato (OPC lineage cells) in the corpus callosum of *Olig1-Cre*; *Setdb1*$^{F/+}$; Rosa26-tomato and *Olig1-Cre*; *Setdb1*$^{F/F}$; Rosa26-tomato mice. n=3 control and mutant mice. (**c**) Quantification of H3K9me3 in OPC lineage cells in corpus callosum of control and mutant mice. Error bars indicate SEM (p<0.001. two-tailed unpaired Student's t test). (**d**) Immunostaining of MBP in the corpus callosum of *Setdb1*$^{F/F}$ and *Olig1-Cre*; *Setdb1*$^{F/+}$ mice at P14. n=3 control and mutant mice. (**e**) Electron micrographs of spinal cord sections from P14 *Olig1-Cre*; *Setdb1*$^{F/+}$ and *Olig1-Cre*; *Setdb1*$^{F/F}$ mice. n=3 control and mutant mice. (**f**) Quantification of myelin sheath thickness (*g*-ratio) in spinal cord. (**g**) In situ hybridization of Plp1 in the spinal cord of *Olig1-Cre*; *Setdb1*$^{F/+}$ and *Olig1-Cre*; *Setdb1*$^{F/F}$ mice at P7. (**h**) Quantification of Plp1 cells in the spinal cord. Error bars indicate SEM (p<0.001. two-tailed unpaired Student's t test). n=3 control and mutant mice. (**i-j**) Immunolabeling of MBP (i) or CC1 (j) in the spinal cord of *Olig1-Cre*; *Setdb1*$^{F/+}$ and *Olig1-Cre*; *Setdb1*$^{F/F}$ mice at P14. n=3 control and mutant mice. (**k**) Quantification of CC1^{+} cells in the spinal cord. Error bars indicate SEM (p<0.001. two-tailed unpaired Student's t test). Scale bars: 50μm in (**a, b** and **d**); 1μm in (**e**); 50μm in (**g** and **i-j**).

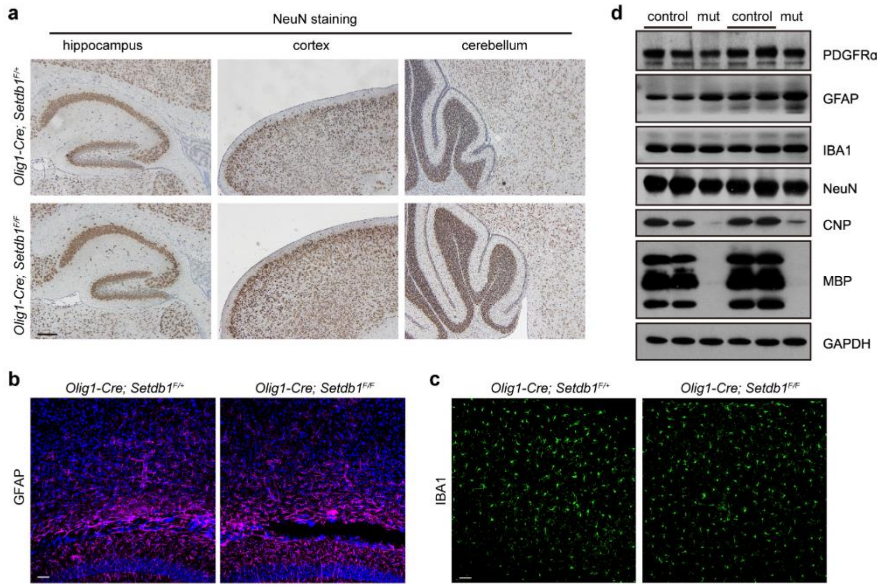

Supplementary Figure 4. SETDB1 ablation in oligodendrocyte lineage does not affect other cell types in brain

(**a**) Immunohistochemical staining of NeuN in the brain of *Olig1-Cre*; *Setdb1*$^{F/+}$ and *Olig1-Cre*; *Setdb1*$^{F/F}$ mice at P14. n=3 control and mutant mice. (**b-c**) Immunostaining of GFAP (b) or IBA1 (c) in the corpus callosum from P14 *Olig1-Cre*; *Setdb1*$^{F/+}$ and *Olig1-Cre*; *Setdb1*$^{F/F}$ mice. n=3 control and mutant mice. (**d**) Western blot analysis of markers of various cells in central nervous system. n=3 independent experiments. Scale bars: 100μm in (**a**); 50μm in (**b-c**).

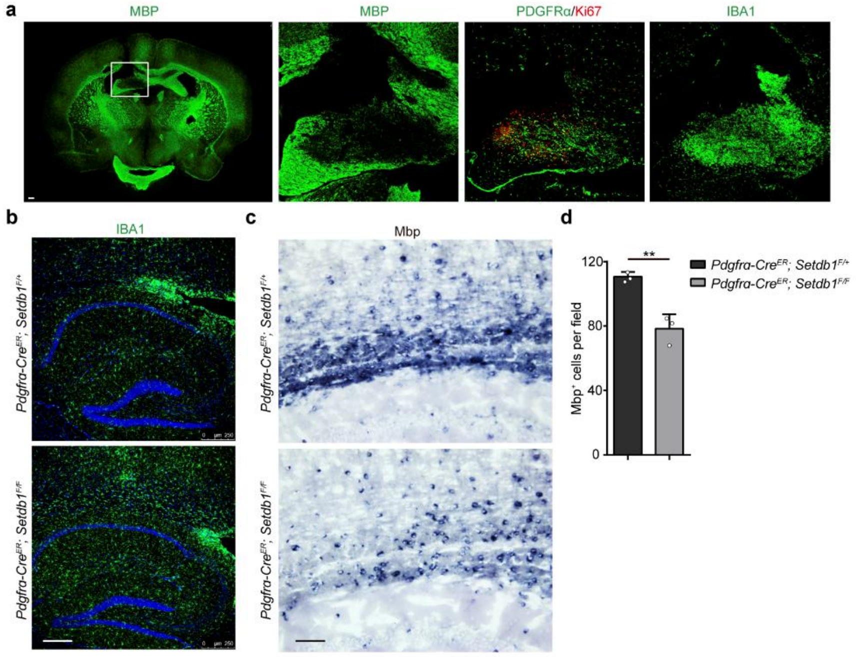

Supplementary Figure 5. SETDB1 is critical for proper myelin repair after demyelination

(**a**) MBP, PDGFRα, Ki67, IBA1 staining shows the LPC lesion pattern in the brain at 7 Dpl. n=3 different mice. (**b**) The IBA1 aggregation scar shows the LPC lesion region in *Pdgfrα-Cre*^{ER}; *Setdb1*$^{F/+}$ and *Pdgfrα-Cre*^{ER}; *Setdb1*$^{F/F}$ mice at 7 Dpl. n=3 control and mutant mice. (**c-d**) In situ hybridization and quantification of Mbp^{+} cell in the lesion regions from *Pdgfrα-Cre*^{ER}; *Setdb1*$^{F/+}$ and *Pdgfrα-Cre*^{ER}; *Setdb1*$^{F/F}$ mice 14 Dpl. Error bars indicate SEM (p=0.0043. two-tailed unpaired Student's t test). n=3 control and mutant mice. Scale bars: 250μm in (**a-b**); 100μm in (**c**).

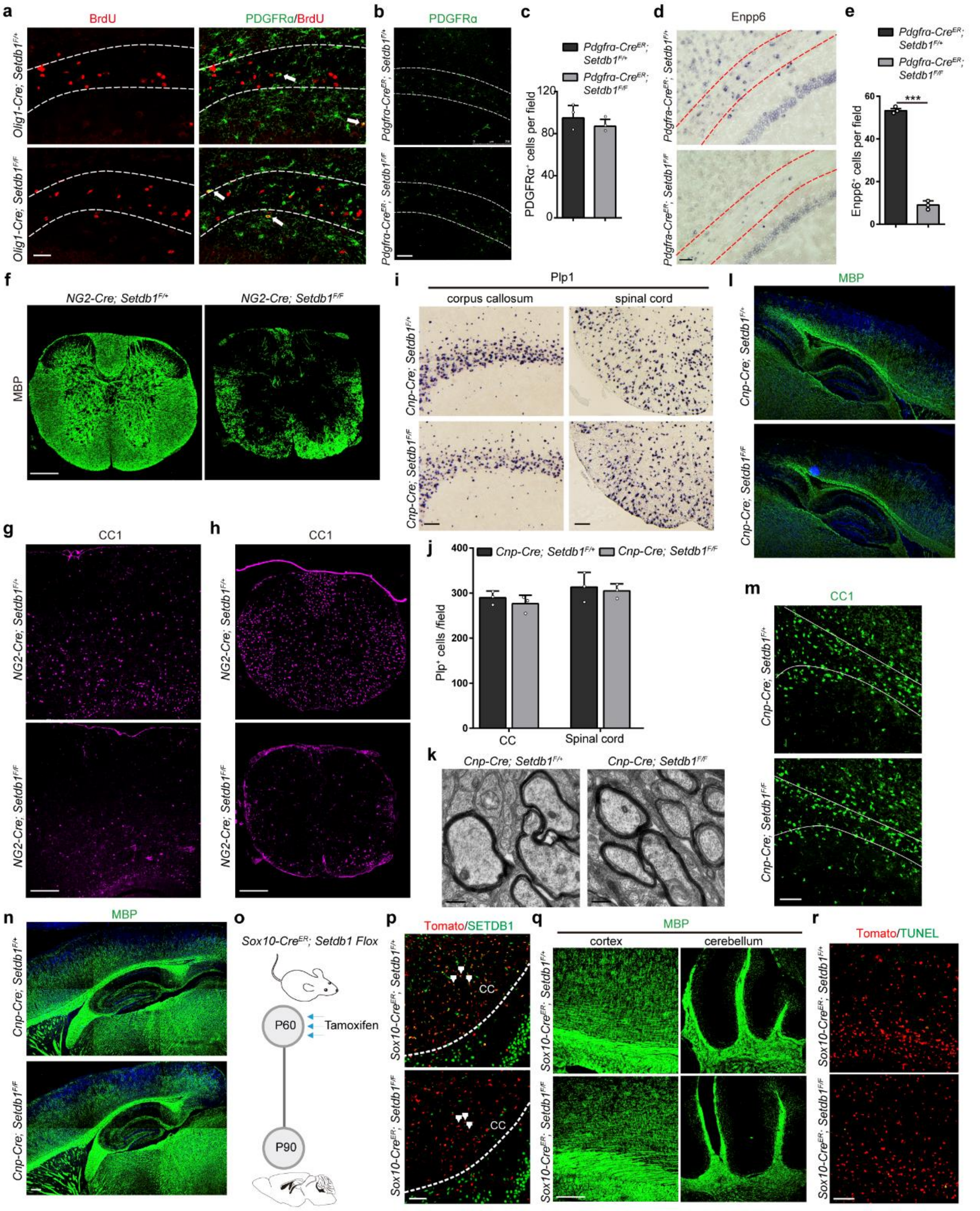

Supplementary Figure 6. OLIG2-SETDB1 repressor complex functions at the onset of OPC differentiation

(**a**) Immunostaining for BrdU and PDGFRα in the corpus callosum from *Olig1-Cre*; *Setdb1*$^{F/+}$ and *Olig1-Cre*; *Setdb1*$^{F/F}$ mice at P14. n=3 control and mutant mice. (**b-c**) Immunostaining and quantification of PDGFRα^{+} cell in the lesion regions from *Pdgfrα-Cre*^{ER}; *Setdb1*$^{F/+}$ and *Pdgfrα-Cre*^{ER}; *Setdb1*$^{F/F}$ mice 14 Dpl. n=3 control and mutant mice. (**d-e**) In situ hybridization and quantification of Enpp6^{+} cell in the corpus callosum from *Olig1-Cre*; *Setdb1*$^{F/+}$ and *Olig1-Cre*; *Setdb1*$^{F/F}$ mice at P14. Error bars indicate SEM (p<0.001. two-tailed unpaired Student's t test). n=3 control and mutant mice. (**f**) Immunostaining of MBP in the spinal cord from *NG2-Cre*; *Setdb1*$^{F/+}$ and *NG2-Cre*; *Setdb1*$^{F/F}$ mice at P14. n=3 control and mutant mice. (**g-h**) Immunostaining of CC1 in the corpus callosum (g) and spinal cord (h) from *NG2-Cre*; *Setdb1*$^{F/+}$ and *NG2-Cre*; *Setdb1*$^{F/F}$

mice at P14. n=3 control and mutant mice. (**i-j**) In situ hybridization and quantification of Plp1^{+} cell in the corpus callosum (left panel) and spinal cord (right panel) of *Cnp-Cre*; *Setdb1*$^{F/+}$ and *Cnp-Cre*; *Setdb1*$^{F/F}$ mice at P14. n=3 control and mutant mice. (**k**) Electron micrographs of transverse optic nerve sections from P14 *Cnp-Cre*; *Setdb1*$^{F/+}$ and *Cnp-Cre*; *Setdb1*$^{F/F}$ mice. n=3 control and mutant mice. (**l-m**) Immunolabeling of MBP (l) or CC1 (m) in the brain of *Cnp-Cre*; *Setdb1*$^{F/+}$ and *Cnp-Cre*; *Setdb1*$^{F/F}$ mice at P14. n=3 control and mutant mice. (**n**) Immunolabeling of MBP in the brain of *Cnp-Cre*; *Setdb1*$^{F/+}$ and *Cnp-Cre*; *Setdb1*$^{F/F}$ mice at P60. n=3 control and mutant mice. (**o**) Schematic diagram showing tamoxifen administration to *Sox10-Cre*^{ER}; *Setdb1 Flox* mice in 3 consecutive days at P60, followed by tissue collection at P90. (**p-q**) Immunolabeling of SETDB1 (p) and MBP (q) in the brain white matter of *Sox10-Cre*^{ER}; *Setdb1*$^{F/+}$; Rosa26-tomato and *Sox10-Cre*^{ER}; *Setdb1*$^{F/F}$; Rosa26-tomato mice at P90. n=3 control and mutant mice. (**r**) TUNEL assay in the corpus callosum of *Sox10-Cre*^{ER}; *Setdb1*$^{F/+}$; Rosa26-tomato and *Sox10-Cre*^{ER}; *Setdb1*$^{F/F}$; Rosa26-tomato mice at P90. n=3 control and mutant mice. Scale bars: 100μm in (**a, b**, **d** and **i**); 250μm in (**f**, **l**, **g**, **h, m**, **n** and **p-r**); 0.5μm in (**k**).

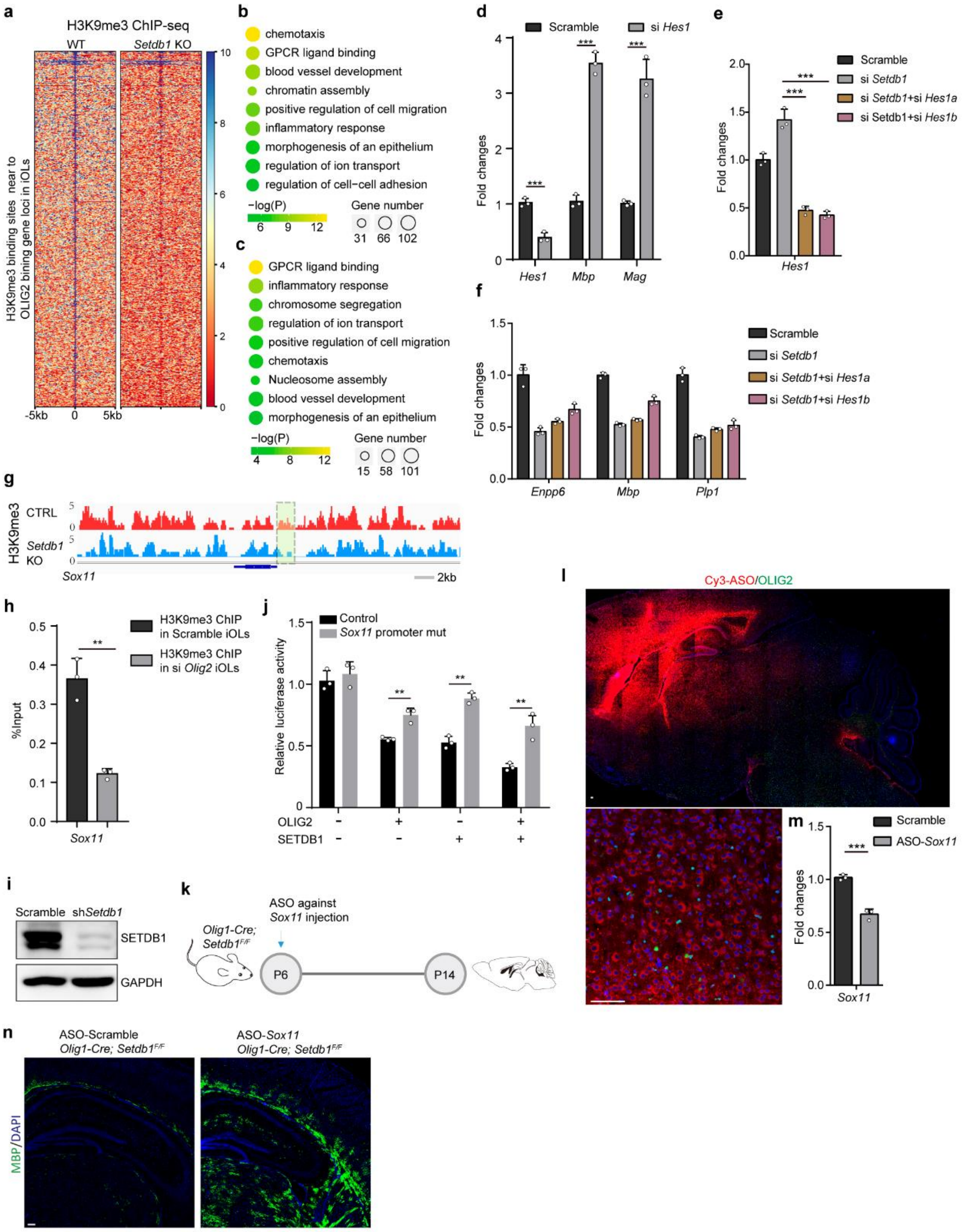

Supplementary Figure 7. OLIG2 recruits SETDB1 to silent inhibitors of oligodendrocyte differentiation

(**a**) Heatmap of H3K9me3 enrichment signals from control and *Setdb1* mutant mouse iOLs around genes occupied with OLIG2 and downregulated from rat OPCs to iOLs. (**b-c**) Representative GO analysis of the significantly upregulated genes between iOLs from *Olig1-Cre*; *Setdb1*$^{F/+}$ and *Olig1-Cre*; *Setdb1*$^{F/F}$ brain cortex (b) and between OPCs under differentiation condition following transfection with *Olig2* and *Olig2-VP64* (c). (**d**) Real-time PCR analysis of indicated genes in rat iOLs transfected with Scramble or siRNA against *Hes1*. Error bars indicate SEM (p<0.001. two-tailed unpaired Student's t test). (**e**) Efficiency of Hes1 knockdown in rat OPCs. Error bars indicate

SEM (***p<0.001. one-way ANOVA). (**f**) Expression of *Enpp6*, *Mbp* and *Plp1* was measured in rat OPCs under differentiation condition following transfection with siRNA against *Setdb1* and *Hes1*. (**g**) Genome browser visualization of H3K9me3-targeting regions on the gene loci of *Sox11* in control and *Setdb1* mutant iOLs. (**h**) H3K9me3 ChIP-qPCR assay on Sox11 promoter in in control and *Olig2* knock-down rat iOLs. Error bars indicate SEM (p=0.0052. two-tailed unpaired Student's t test). (**i**) Efficiency of Setdb1 knockdown in 293T was examined by Western Blot. n=3 independent experiments. (**j**) Luciferase activity in 293T cells transfected with luciferase reporter driven by wild-type or mutant *Sox11* promoter together with OLIG2 or SETDB1. Error bars indicate SEM (OLIG2+, p=0.0012; SETDB1+, p=0.0029; OLIG2+ SETDB1+, p=0.0046; two-tailed unpaired Student's t test). (**k**) Schematic diagram showing modified ASO to *Sox11* injected into *Olig1-Cre*; *Setdb1*$^{F/F}$ pups at P6 and followed by tissue collection at P14. (**l**) Immunolabeling of OLIG2 and Cy3 fluorescence tagged ASO showed the efficiency in mouse forebrain. n=3 different mice. (**m**) Efficiency of *Sox11* knockdown by ASO in the whole forebrain as shown. Error bars indicate SEM (p<0.001. two-tailed unpaired Student's t test). (**n**) Immunolabeling for MBP on the corpus callosum from ASO-Scramble, *Olig1-Cre*; *Setdb1*$^{F/F}$ and ASO-si *Sox11*, *Olig1-Cre*; *Setdb1*$^{F/F}$ mice at P14. n=3 control and mutant mice. Scale bars: 50μm in (**l**); 100μm in (**n**).
