# Supplementary material for: The Oligodendrocyte Transcription Factor 2 OLIG2 regulates transcriptional repression during myelinogenesis in rodents
Source: Nat Commun. 2022 Mar 17;13:1423. doi: 10.1038/s41467-022-29068-z (PMC8931116; doi:10.1038/s41467-022-29068-z)
Supplement: Supplementary file 2 — Reporting Summary [file 41467_2022_29068_MOESM2_ESM.pdf]

nature research

Corresponding author(s): Wei Mo
Last updated by author(s): Jan 24, 2022

Reporting Summary

Nature Research wishes to improve the reproducibility of the work that we publish. This form provides structure for consistency and transparency in reporting. For further information on Nature Research policies, see our Editorial Policies and the Editorial Policy Checklist.

Statistics

For all statistical analyses, confirm that the following items are present in the figure legend, table legend, main text, or Methods section.

n/a	Confirmed	
☐	☒	The exact sample size (n) for each experimental group/condition, given as a discrete number and unit of measurement
☐	☒	A statement on whether measurements were taken from distinct samples or whether the same sample was measured repeatedly
☐	☒	The statistical test(s) used AND whether they are one- or two-sided<br>*Only common tests should be described solely by name; describe more complex techniques in the Methods section.*
☐	☒	A description of all covariates tested
☒	☐	A description of any assumptions or corrections, such as tests of normality and adjustment for multiple comparisons
☐	☒	A full description of the statistical parameters including central tendency (e.g. means) or other basic estimates (e.g. regression coefficient) AND variation (e.g. standard deviation) or associated estimates of uncertainty (e.g. confidence intervals)
☐	☒	For null hypothesis testing, the test statistic (e.g. F, t, r) with confidence intervals, effect sizes, degrees of freedom and P value noted<br>*Give P values as exact values whenever suitable.*
☒	☐	For Bayesian analysis, information on the choice of priors and Markov chain Monte Carlo settings
☒	☐	For hierarchical and complex designs, identification of the appropriate level for tests and full reporting of outcomes
☒	☐	Estimates of effect sizes (e.g. Cohen's d, Pearson's r), indicating how they were calculated

Our web collection on statistics for biologists contains articles on many of the points above.

Software and code

Policy information about availability of computer code

Data collection	Zen 2.3 blue, black editions, ZEISS Imager. Z2, Leica SP8 and Las X 2.0.1 were used to collect Immunohistochemical data; Hitachi HT-7800 transmission electron microscopy was used to collect data from transmission electron microscopy; The CFX Connect Real-Time PCR Detection System was used to collect data from RT–qPCR, Biosystems c280 was used to collect western blot data; Illumina sequencer Hiseq-Xten PE150 was used to collect sequencing data.
Data analysis	Statistical analyses were performed with the GraphPad Prism 7.0 software. RNA-seq raw data were initially filtered to obtain clean data after quality control. Clean data were aligned to the mouse genome (mm10) or rat genome (rn6) by HISAT2-2.1.0. Raw counts for each gene were calculated by Htseq-0.7.2. StringTie-1.3.0 was used to estimate the expression level of detected genes. DEGs were defined as genes with FDR less than 0.001 and fold change larger than 1.5. The identification of ChIP-seq peaks was performed using HOMER-v4.11. Annotated positions for promoters, exons, introns and other features were based on RefSeq transcripts and repeat annotations from the University of California, Santa Cruz. The sequencing reads were aligned to the rat genome UCSC build rn6 by using Bowtie2-2.1.0

For manuscripts utilizing custom algorithms or software that are central to the research but not yet described in published literature, software must be made available to editors and reviewers. We strongly encourage code deposition in a community repository (e.g. GitHub). See the Nature Research guidelines for submitting code & software for further information.

Data

Policy information about availability of data

All manuscripts must include a data availability statement. This statement should provide the following information, where applicable:

- Accession codes, unique identifiers, or web links for publicly available datasets
- A list of figures that have associated raw data
- A description of any restrictions on data availability

The raw sequencing data generated in this study have been deposited in the National Center for Biotechnology Information (NCBI) BioProject database under BioProject: PRJNA673758 [https://.ncbi.nlm.nih.gov/bioproject]. The reference mouse genome was downloaded from database: http://hgdownload.cse.ucsc.edu/goldenPath/mm10/bigZips/. The reference rat genome was downloaded from database: http://hgdownload.cse.ucsc.edu/goldenPath/rn6/bigZips/

Field-specific reporting

Please select the one below that is the best fit for your research. If you are not sure, read the appropriate sections before making your selection.

- [x] Life sciences
- [ ] Behavioural & social sciences
- [ ] Ecological, evolutionary & environmental sciences

For a reference copy of the document with all sections, see nature.com/documents/nr-reporting-summary-flat.pdf

Life sciences study design

All studies must disclose on these points even when the disclosure is negative.

Sample size	No sample-size calculations were performed. Sample size was determined to be adequate based on the magnitude and consistency of measurable differences between groups. Sample size were chosen based on 80% statistical test power and 0.05 significance level.
Data exclusions	On principle, data were only excluded for failed experiments, reasons for which included suboptimal activation and microbial contamination
Replication	At least 3 independent replicates were performed for each experiment.
Randomization	No randomization of mice. Mice analyzed were litter mates and sex-matched whenever possible. Samples other than those involving in mice were allocated into experimental groups at random.
Blinding	Investigators were not blinded to mouse genotypes during experiments, because we need to check the genotype of mice before experiments. Data reported for mouse experiments are not subjective. For experiments other than those involving in mice, investigators were blinded to allocation.

Reporting for specific materials, systems and methods

We require information from authors about some types of materials, experimental systems and methods used in many studies. Here, indicate whether each material, system or method listed is relevant to your study. If you are not sure if a list item applies to your research, read the appropriate section before selecting a response.

Materials & experimental systems

n/a	Involved in the study	
[ ]	[x]	Antibodies
[x]	[ ]	Eukaryotic cell lines
[x]	[ ]	Palaeontology and archaeology
[ ]	[x]	Animals and other organisms
[x]	[ ]	Human research participants
[x]	[ ]	Clinical data
[x]	[ ]	Dual use research of concern

Methods

n/a	Involved in the study	
[ ]	[x]	ChIP-seq
[x]	[ ]	Flow cytometry
[x]	[ ]	MRI-based neuroimaging

Antibodies

Antibodies used	For western blotting: rabbit anti-SETDB1 (Proteintech, 1:3000, Cat#11231-1-AP), rabbit anti-OLIG2 (talent biomedical, 1:3000, Cat#AP0337), mouse anti-HA probe (Santa Cruz Biotechnology, 1:10000, Cat#SC-7392), rabbit anti-PDGFRα (Santa Cruz Biotechnology, 1:3000, Cat#sc338), mouse anti-GFAP (talent biomedical, 1:3000, Cat#AM0123), rabbit anti-IBA1 (WAKO, 1:3000, Cat#019-19741), mouse anti-NeuN (EMD Millipore, 1:3000, Cat#mab377), mouse anti-CNP (EMD Millipore, 1:3000, Cat#MAB326) , goat anti-MBP (Santa Cruz Biotechnology, 1:3000, Cat#sc-13914), mouse anti-GAPDH (Santa Cruz Biotechnology, 1:3000, Cat#365062), rabbit anti-EHMT1 (ABclonal Technology, 1:3000, Cat#A8513), rabbit anti-EHMT2 (ABclonal Technology, 1:3000, Cat#A1247), rabbit anti-PRDM2 (ABclonal Technology, 1:3000, Cat#A13157), rabbit anti-NKX2-2 (ABclonal Technology, 1:3000, Cat#A16696), rabbit anti-YY1 (ABclonal Technology, 1:3000, Cat#A19569), rabbit anti-SOX10 (ABclonal Technology, 1:3000,

	Cat#A8658), rabbit anti-ZFP191 (ABclonal Technology, 1:3000, Cat#A7500),For Immunohistochemistry: mouse anti-CNP (EMD Millipore, 1:1000, Cat#MAB266), goat anti-MBP (Santa Cruz Biotechnology, 1:1000, Cat#sc-13914), mouse anti-SETDB1 (Thermofisher, 1:500, Cat#MA5-15722), mouse anti-BrdU (Abcam, 1:1000, Cat#ab8039), rabbit anti-PDGFRα (Santa Cruz Biotechnology, 1:500, Cat#sc338), rabbit anti-GSTpi (MBL, 1:200, Cat#311), mouse anti-H3K9me3 (Abcam, 1:1000, Cat#ab8898), mouse anti-NeuN (EMD Millipore, 1:1000, Cat#mab377), mouse anti-GFAP (talent biomedical, 1:500, Cat#AM0123), rabbit anti-IBA1 (WAKO, 1:500, Cat#019-19741), mouse anti-KI67 (BD BIOSCIENCES, 1:1000, Cat#550609); For ChIP: rabbit anti-H3K9me3 (Abcam, Cat#ab8898), rabbit anti-OLIG2 (EMD Millipore, Cat#ab9610), mouse anti-HA probe (Santa Cruz Biotechnology, Cat#SC-7392); For Co-IP: rabbit anti-OLIG2 (EMD Millipore, Cat#ab9610), mouse anti-OLIG2 (EMD Millipore, Cat#MABN50), rabbit anti-SETDB1 (Proteintech, Cat#11231-1-AP). For OPC culture: anti-O4 antibody and anti-A2B5 antibody were home-made by collecting the supernatant of hybridoma
Validation	Antibodies are commercial and were validated by manufacturers for indicated species and applications. Such as: rabbit anti-SETDB1 (PMID: 30692625), mouse anti-HA probe (PMID: 29069680), rabbit anti-PDGFRα (PMID: 27242400), mouse anti-GFAP (PMID:3003434), mouse anti-NeuN (PMID:3003434), goat anti-MBP (PMID:26311780), mouse anti-GAPDH (PMID: 34408754), rabbit anti-EHMT1 (https://abclonal.com.cn/catalog/A8513,), rabbit anti-EHMT2 (https://abclonal.com.cn/catalog/A1247), rabbit anti-PRDM2 (https://abclonal.com.cn/catalog/A13157), rabbit anti-NKX2-2 (https://abclonal.com.cn/catalog/A16696), rabbit anti-YY1 (https://abclonal.com.cn/catalog/A19569), rabbit anti-SOX10 (https://abclonal.com.cn/catalog/A8658), rabbit anti-ZFP191 (https://abclonal.com.cn/catalog/A7500), mouse anti-CNP (PMID:21613483), mouse anti-BrdU (PMID:29321633), rabbit anti-GSTpi (PMID: 23440860), mouse anti-H3K9me3 (PubMed: 28807015) rabbit anti-IBA1 (PMID:22956822), mouse anti-KI67 (PMID: 19703988) rabbit anti-OLIG2 (PMID: 21459827)

Animals and other organisms

Policy information about studies involving animals; ARRIVE guidelines recommended for reporting animal research

Laboratory animals	Description of research mice and rats used for experiments can be found in the relevant figure legends and Methods.
Wild animals	The study did not involve wild animals.
Field-collected samples	The study did not involve samples collected from the field.
Ethics oversight	All mouse and rat experiments in this study were approved by Institutional Animal Care and Use Committee and were performed in accordance with animal practice, which defined by the Xiamen University Laboratory Animal Center.

Note that full information on the approval of the study protocol must also be provided in the manuscript.

ChIP-seq

Data deposition

☒ Confirm that both raw and final processed data have been deposited in a public database such as GEO.

☒ Confirm that you have deposited or provided access to graph files (e.g. BED files) for the called peaks.

Data access links<br>*May remain private before publication.*	https://www.ncbi.nlm.nih.gov/bioproject/?term=PRJNA673758
Files in database submission	Input of Rattus norvegicus<br>Input of Rattus norvegicus<br>H3K9me3 ChIP-seq of Rattus norvegicus: iOL-1<br>H3K9me3 ChIP-seq of Rattus norvegicus: OPC-1<br>Olig2 ChIP-seq of Rattus norvegicus: iOL-1<br>Olig2 ChIP-seq of Rattus norvegicus: OPC-1<br>HA-Setdb1 ChIP-seq of Rattus norvegicus: OPC-1<br>HA-Setdb1 ChIP-seq of Rattus norvegicus: iOL-1<br>Input of Mus musculus-1<br>H3K9me3 ChIP-seq of Mus musculus: WT-iOL-1<br>H3K9me3 ChIP-seq of Mus musculus:Mut- iOL-1
Genome browser session<br>(e.g. UCSC)	No longer applicable

Methodology

Replicates	ChIP-seq and analysis subsection
Sequencing depth	Library concentrations were mixed equally for sequencing at Hiseq-Xten to generate 150 bp reads from paired-end.
Antibodies	anti-H3K9me3 (Abcam, Cat#ab8898)<br>anti-OLIG2 (EMD Millipore, Cat#ab9610)<br>anti-HA probe (Santa Cruz Biotechnology, Cat#SC-7392)

Peak calling parameters	Clean reads were aligned to the mouse genome (mm10) and rat genome (rn6) using Bowtie2 (version 2.2.8). Enriched peaks were identified by HOMER v4.10.1 with histone style (http://homer.ucsd.edu/homer).
Data quality	Peaks were called using an input control. Peaks were called at an FDR of 0.1% and required 4-fold enrichment over the input control.
Software	bowtie2; homer; fastp0.19.0; deepTools2.0; ComputeMatrix; PlotHeatmap
