# Supplementary material for: The Oligodendrocyte Transcription Factor 2 OLIG2 regulates transcriptional repression during myelinogenesis in rodents
Source: Nat Commun. 2022 Mar 17;13:1423. doi: 10.1038/s41467-022-29068-z (PMC8931116; doi:10.1038/s41467-022-29068-z)
Supplement: Supplementary file 4 — Description of Additional Supplementary Files [file 41467_2022_29068_MOESM4_ESM.docx]

Description of Additional Supplementary Files

Title: Supplementary Video 1

Description: Myelin-deficient phenotypes of Setdb1 mutant mouse
